# Toxicity of Water-Soluble Fraction in *Echinometra lucunter* for the 2019 and 2021 Accidental Oil Spills on the Brazilian Coast

**DOI:** 10.1007/s00128-026-04247-6

**Published:** 2026-04-28

**Authors:** Juliana dos Santos Pereira, Debora Cristina Nascimento de Santana, Iara Costa Souza, Rafael Garrett Dolatto, Marco Tadeu Grassi, Magdalena V. Monferrán, João Henrique Alliprandini da Costa, Pedro Henrique Cipresso Pereira, Camilo Dias Seabra Pereira, Helen Sadauskas-Henrique

**Affiliations:** 1 Laboratório de Ecofisiologia e Bioquímica de Organismos Aquáticos (LEBIO), Universidade Santa Cecília (UNISANTA), Rua Oswaldo Cruz, 277 (Boqueirão), Santos, SP 11045-907 Brazil; 2 Universidade Estadual Paulista, São Vicente, SP Brazil; 3 Departamento de Ciências Fisiológicas, Universidade Federal de São Carlos, São Carlos, SP Brazil; 4 Grupo de Química Ambiental, Universidade Federal do Paraná, Centro Politécnico, Curitiba, PR Brazil; 5 Departamento de Bioquímica Clínica, Facultad de Ciencias Químicas, Universidad Nacional de Córdoba, y CONICET, CIBICI, Ciudad Universitaria, Córdoba, Argentina; 6 Laboratório de Biologia de Organismos Marinhos e Costeiros, Universidade Santa Cecília, Santos, SP Brazil; 7 Projeto Conservação Recifal, Recife, Pernambuco Brazil; 8 Laboratório de Ecotoxicologia, Universidade Santa Cecília, Santos, SP Brazil; 9 Departamento de Ciências do Mar, Universidade Federal de São Paulo, Santos, SP Brazil; 10 Departamento de Ciências Fisiológicas, Universidade Federal de São Carlos (DCF/UFSCar), Rod. Washington Luiz, Km 235, São Carlos, SP 13565-905 Brazil

**Keywords:** Weathered oil, Toxicity, Early life stages, PAHs, Metals

## Abstract

The present study evaluated the embryonic–larval development of sea urchins (*Echinometra lucunter*) exposed to water-soluble fractions (WSF) of oil spilled on the Brazilian coast in 2019 and recovered post-accident in 2021. In 2019, organisms were exposed to WSF concentrations of 35.01%, 45.51%, 59.17%, 76.9%, and 100%. In 2021, the concentrations tested were 0.88%, 3.30%, 9.42%, 20.71%, 35.01%, 45.51%, 59.17%, 76.9%, and 100%. The EC_50_-42 h, NOEC, and LOEC were determined, and the concentrations of PAHs and metal(oid)s were analyzed in the 100% WSF. 2019 PAH levels were above regulatory limits, whereas all 16 analyzed PAHs were below detection limits in 2021. Metal(oid) concentrations above regulatory limits were observed for Cd, Cr, Cu, Ni, Pb, Se, Zn, and Au in 2019, and for Fe, Cu, and Se in 2021. Trace elements of emerging concern, for which no safe limits have been established in Brazilian legislation, were detected in both WSF. The EC_50_-42 h, NOEC, and LOEC indicated reduced toxicity in 2021 (65.42%, 35.01%, and 45.51%, respectively) compared to 2019 (39.45%, > 35.01%, and 35.01%, respectively), with total inhibition of embryo–larval development at 100% WSF in both years. This reduction in toxicity may be related to the absence of detectable PAHs and to changes in the concentrations of specific metal(oid)s in the post-accident WSF.

**Supplementary Information:**

The online version contains supplementary material available at 10.1007/s00128-026-04247-6.

## Introduction

Over the years, accidents related to oil transportation in both freshwater and marine environments (Lobão et al. 2022; Sadauskas-Henrique et al. 2021, 2024) have become increasingly frequent worldwide. One of the most significant incidents in Brazil was the spill of over 5.000 tons of crude oil along the beaches of the Northeastern coast in 2019 (Soares et al. 2020). Studies have indicated that 44 Marine Protected Areas were affected within their boundaries by the oil slicks (Nunes et al. 2023). Among the most impacted regions was the Marine Protected Area Costa dos Corais (MPACC), Brazil’s largest federally protected marine area, spanning the Northeastern states of Alagoas and Pernambuco (Nunes et al. 2023). According to Lessa et al. (2021), this environmental disaster was characterized by the presence of oil slicks in over a thousand locations across nine Northeastern states (Alagoas, Bahia, Ceará, Maranhão, Paraíba, Pernambuco, Piauí, Rio Grande do Norte, and Sergipe), as well as in Espírito Santo and Rio de Janeiro. The absence of effective coordination and clear guidelines from the Brazilian federal government hindered an immediate response, exacerbating delays in governmental actions to mitigate the spill. This inefficiency was primarily due to the dissolution of the executive and support committees responsible for responding to oil spill incidents, including the Contingency Plan for Oil Pollution team in early 2019 (Government of Brazil 2019). Furthermore, the weakening of environmental policies (Abessa et al. 2019; Soares et al. 2020), budget cuts in research funding, and the COVID-19 pandemic further compromised the ability of Brazilian institutions to assess and mitigate the impacts of this uncontrolled environmental disaster. The oil contamination reached the beaches, severely affecting local communities and marine organisms, primarily causing mortality through asphyxiation (Araújo et al. 2020; Magris and Giarrizzo 2020).

The physicochemical characteristics of oil are specific to each region. Lourenço et al. (2020) reported that crude oil samples collected from beaches along the Northeastern coast were denser than seawater. Studies have indicated that the chemical characteristics of the oil are similar to those of Venezuelan oil, suggesting that it was either heavily weathered or derived from heavy crude oils (Oliveira et al. 2020). Despite the oil identification not being established, Santana et al. (2025) showed that the oil fragments, from the same oil spill, collected on the beaches of Bahia, were a source of hydrocarbons to the water column via water-soluble fraction (WSF). Also, these authors found light aliphatic hydrocarbons in the WSF, which, together with the polycyclic aromatic hydrocarbons (PAHs), indicate minimal weathering processes (e.g., evaporation, dissolution, biodegradation, and degradation) as the material was transported to the shore.

Crude oil is a complex mixture of thousands of distinct molecules, and various processes occur concurrently when oil interacts with seawater, like evaporation, dissolution, emulsion, photolysis, and biodegradation (Liu et al. 2012; Sadauskas-Henrique et al. 2021). The WSF of petroleum is particularly toxic to aquatic organisms, as it contains a high concentration of aromatic hydrocarbons and their homologs (Liu et al. 2012) and metals (Zhang et al. 2020). The water-accommodated fraction (WAF) includes the WSF and oil droplets (Liu et al. 2022). Both fractions can induce diverse toxicological effects on marine species (Stubblefield et al. 2023). Among the most harmful components of the petroleum WSF are PAHs. The United States Environmental Protection Agency (U.S. EPA) prioritized 16 PAHs due to their toxicity and persistence in aquatic environments (Sadauskas-Henrique et al. 2021). Although they occur in low concentrations, the PAHs are the main substances causing toxicity. Having hydrophobic characteristics, they can be transported to cells and metabolized by enzymes such as P450 (CYP), thus presenting a great carcinogenic capacity (Sadauskas-Henrique et al. 2021).

Some of these compounds exhibit relative solubility and consequent bioavailability in water, rendering marine organisms more vulnerable as they absorb these contaminants through tissues and directly ingest contaminated water or food (Sadauskas-Henrique et al. 2021). Metal(oids) are elements that also occur in crude oils, though not numerous in terms of a metal variety (mostly Ni, V, and Fe) and abundant in unprocessed crude oil (Maryutina and Timerbaev 2017). Once bioavailable, metal(oid) can suffer several chemical processes (e.g., complexation, redox reactions, and sorption) that result in their transformation into different forms, such as free hydrated ions (M^2+^), organic and inorganic complexes. It may remain in the solid phase as suspended particles or precipitate. All these chemical forms interact with aquatic organisms, potentially inducing biological disturbances (Wang et al. 2014).

In this context, toxicity assays are commonly employed to assess the adverse effects on aquatic organisms. These assays are based on the dose-response principle, facilitating the acquisition of short-, medium-, or long-term responses (Bittencourt et al. 2018). Testing periods are generally 24 and 96 h. Toxicological tests provide an approximate idea of exposure in a realistic environment (Gissi et al. 2021).

Benthic, territorial, and low-mobility animals are ideal bioindicators. Among them, the sea urchin *Echinometra lucunter* Linnaeus, 1758 (Echinodermata: Echinoidea) is widely used due to several advantages, such as its geographical distribution, abundance, ease of sampling, handling, laboratory maintenance, gamete collection, and external fertilization (Gonzalez-Aravena et al. 2015). Furthermore, due to its sensitivity and ecological relevance, *Echinometra lucunter* has become a model organism in toxicity assessments, with the embryonic-larval development test being widespread for such evaluations (Santana et al. 2025). This test has been well established through standard protocols (ABNT nº15350 2023).

With this background, we hypothesized that the oil recovered shortly after the spill would be more toxic than the oil recovered 2 years later, due to weathering and degradation. To test this hypothesis, we evaluated the toxic effects of oil collected in 2019 and 2021 from the Northeastern coast of Brazil by: (i) determining the concentrations of PAHs and metal(oid)s in the 100% WSF of the oil, and (ii) assessing the toxicity of the WSF through embryo–larval development tests with *E. lucunter*.

## Materials and Methods

### Water-Soluble Fractions Preparations

Natural seawater from Guarujá (SP, Brazil) was used, filtered (0.45 μm Millipore^®^ membrane), aerated, maintained at 24 °C, with salinity ranging from 33 to 37 ppt (KAVI refractometer) and pH from 7.8 to 8.4 (Oakton Bench 700) (ABNT 2023). Oil samples, collected by divers in 2019 and 2021 from Japaratinga Beach (MPACC, AL, Brazil) and stored at − 20 °C, were used to prepare the WSF following NBR 15,469 (ABNT 2021). For the 100% WSF (from 2019 to 2021), the oils were mixed with seawater at a 1:9 ratio (water: oil), magnetically stirred for 20 h (vortex reaching 1/3 of the water column height), allowed to settle for 1 h, and the liquid phase was filtered (0.45 μm) for immediate use as the stock solution.

From the 100% WSF, dilutions were prepared using a 1:3 dilution factor, resulting in the following concentrations: 35.01%, 45.51%, 59.17%, 76.9%, and 100% (2019 oil); and 0.88%, 3.30%, 9.42%, 20.71%, 35.01%, 45.51%, 59.17%, 76.9%, and 100% (2021 oil). For the tests, 2.5 mL of each WSF concentration (filtered, ABNT 2021) was distributed into five test tubes per concentration, following the microscale testing procedure described by Nilin et al. (2008).

### PAHs Extraction and Analysis in the 100% Water-Soluble Fractions

Polycyclic Aromatic Hydrocarbons (PAHs) were extracted from the 100% WSF using a vortex-assisted dispersive liquid-liquid extraction (VA-DLLME) protocol, as described by Dolatto et al. (2023). Subsequent quantification of PAHs was performed using a gas chromatograph coupled with a triple quadrupole mass spectrometer (GC-MS/MS, Shimadzu^®^ model QP2010-TQ8040). Quality assurance and control (QA/QC) measures were implemented, including fortification tests with high-purity PAHs analytical standards obtained from Merck/Sigma-Aldrich^®^. Analytical curves were established through external calibration using five deuterated internal standards, demonstrating appropriate linearity (determination coefficients, r^2^ ≥ 0.99 for all 16 analytes and the surrogate standard) within the selected linear range of 0.1–2 µg/L (Supplementary Material, S1). Limits of detection (LOD) and quantification (LOQ) were calculated in accordance with established literature (Dolatto et al. 2023) and are presented in the Supplementary Material (S2). The analytical protocol’s precision and accuracy were validated through triplicate recovery tests, where samples spiked with PAHs at a concentration of 1.0 µg/L yielded recovery values ranging from 84.1 to 107.8%, with uncertainties consistently below 12.9%. Furthermore, each extraction batch was monitored using p-Terphenyl-D14 (1.0 µg L^−1^) as a surrogate standard, which showed an average recovery of 84.2% with a relative standard deviation (RSD) below 11.9%. Analysis of blank samples (ultrapure water) confirmed that PAH concentrations were below the method’s LOD, ensuring no significant background contamination.

### Multi-elemental Analysis in the 100% Water-Soluble Fraction

Element quantification was performed using an inductively coupled plasma mass spectrometer (Q-ICPMS, Agilent 7500 Series CX technology), equipped with an ASX-100 auto-sampler (CETAC Technologies, Omaha, NE, USA). The analyzes were conducted at the Food Science and Technology Institute of Córdoba (YCITAC-Universidad Nacional de Córdoba, Argentina). The concentrations of elements present in the 100% WSF were measured in triplicate to ensure accuracy and reproducibility. Certified reference materials (NIST 1640a and NIST 1643e) were used for analytical calibration and quality control. The average recovery was 108 ± 9%. Limits of quantification (LOQ) and detection (LOD) for the metals analysis of the 2019 and 2021 oil WSF are presented in the supplementary material (S3).

### Organism Collection and Gamete Acquisition

Sea urchins *E. lucunter* (*n* = 29) were collected in November by snorkeling at Palmas Island (São Paulo, Brazil). During transportation in a thermal box with aerated natural seawater (pH 8.2; salinity: 35 ppt; temperature: 27 °C) to prevent gamete release, they were acclimated for 24 h and subsequently maintained in tanks with natural seawater under controlled conditions. Gamete acquisition followed NBR 15,350 (ABNT 2023): release was induced by injecting 1 mL of KCl (0.5 M) into the perioral region. Female gametes were collected by inverting the animal over a beaker with filtered seawater, and male gametes were collected directly from the gonopores into an ice-chilled beaker. The fertilization rate was assessed microscopically (400×) to ensure ≥ 90% success.

### Toxicity Assays

Toxicity assays were conducted following standard procedures (ABNT 2023) and adapted for microscale testing (Nilin et al. 2008). Four replicates were prepared for each WSF concentration, with an additional replicate for measuring physical-chemical parameters (pH, salinity, dissolved oxygen, temperature) and for PAHs and multi-elemental analyses. Each test tube contained 2.5 mL of the test solution and 65.5 µL of seawater, corresponding to 300 newly fertilized embryos. The tests were conducted under static conditions for 42 h, with a 16:8 h light: dark photoperiod, at a constant temperature of 26.2 ± 2 °C. After this period, 0.05 mL of borax-buffered formalin was added for larval fixation. The percentage of larval development (normal larvae at the pluteus stage versus abnormal larvae at earlier stages such as morula, blastula, and gastrula; ABNT 2023) was assessed in a Sedgwick–Rafter chamber under an optical microscope (400×), counting the first 100 organisms from each replicate. The assay was valid when ≥ 80% of control larvae exhibited normal development within 42 h.

### Potential Sources of Metal(oid)s in the Water-Soluble Fraction

To evaluate potential sources of metal(oid)s in the WSF, we generated scatterplots of individual metal concentrations against Al and Fe, which are widely recognized as lithogenic tracers. Concentrations were obtained from the elemental analysis presented in Table 2. Metal(oid)s with values above detection limits were selected for each sampling year (2019 and 2021), excluding Al and Fe. Scatterplots were constructed using a log-log scale to visualize trace elements and major constituents simultaneously. Metal(oid)s showing proportionality with Al or Fe were interpreted as predominantly associated with natural terrigenous inputs, whereas deviations from these tracers indicated possible anthropogenic enrichment (Supplementary Material, S4). 2019 scatterplots could not be produced because Al and Fe were below detection limits.

### Statistical Analyses

All data were expressed as the mean ± standard error (*n* = 4), with statistical significance set at *p* < 0.05. Data were tested for normality using the Shapiro-Wilk test and for homogeneity of variances using Levene’s test. Differences among treatment groups were assessed using a one-way analysis of variance (ANOVA), followed by the Holm-Sidak post hoc test. All statistical analyses and graphical representations were performed using Sigma Stat and Sigma Plot software (Jandel Scientific, San Jose, USA). The median effective concentration (EC_50_), corresponding to the concentration inducing a specific quantal effect (lethal or sublethal) in 50% of exposed organisms, was determined (ABNT 2023).

## Results

### Physical-Chemical Analysis of Test Water

For the tests with oil recovered in 2019, pH values ranged from 7.98 to 8.16 (8.08 ± 0.08), dissolved oxygen ranged from 6.42 to 6.56 mg L^−1^ (6.47 ± 0.10), and salinity from 32 to 33 ppt (32.8 ± 2). For the tests with oil recovered in 2021, pH values ranged from 8.06 to 8.34 (8.15 ± 0.13), dissolved oxygen from 6.17 to 6.46 mg L^−1^ (6.39 ± 0.057), and salinity from 32 to 33 ppt (32.85 ± 1.85).

### Analysis of Polycyclic Aromatic Hydrocarbons (PAHs) in the 100% WSF of Oil Recovered in 2019 and 2021

The concentration of the 16 priority PAHs analyzed in the 100% WSF is presented in Table 1. The PAHs exhibit concentrations ranging from 109 to 1963 ng/L. The order of PAHs concentrations, from lowest to highest, is as follows: acenaphthylene < acenaphthene < naphthalene < fluoranthene < benzo[k]fluoranthene < fluorene < benzo[b]fluoranthene < benzo[a]pyrene < benzo[a]anthracene < benzo[g,h,i]perylene < chrysene < indeno[1,2,3-cd]pyrene < pyrene < dibenzo[a,h]anthracene < anthracene < phenanthrene. The PAHs with concentrations above 1 µg/L were phenanthrene, anthracene, and dibenzo[a,h]anthracene. In contrast, the oil sample recovered in 2021 exhibited concentrations below the detection limit for all 16 PAHs (Table 1).

**Table 1:** Concentration (ng/L) of polycyclic aromatic hydrocarbons (PAHs) in the 100% WSF used in the chronic test of oils recovered in the Marine Protected Area Costa dos Corais in 2019 and 2021

	2019	2021	CONAMA 357/2005
			Conservation units
*Low molecular weight*		ng/L	
Naphthalene	146	< LOD	–
Acenaphthylene	109	< LOD	–
Acenaphthene	129	< LOD	–
Fluorene	226	< LOD	–
Phenanthrene	1963	< LOD	–
∑PAHs LMW	**2573**	< LOD	–
*High molecular weight*			
Anthracene	1890	< LOD	–
Fluoranthene	148	< LOD	–
Pyrene	755	< LOD	–
Benz[a]anthracene	381	< LOD	18
Chrysene	437	< LOD	18
Benzo[b]fluoranthene	290	< LOD	18
Benzo[k]fluoranthene	155	< LOD	18
Benzo[a]pyrene	356	< LOD	18
Indeno[1,2,3-cd]pyrene	616	< LOD	18
Dibenz[ah]anthracene	1037	< LOD	18
Benzo[ghi]perylene	404	< LOD	–
∑PAHs HMW	**6469**	< LOD	**90.2**
∑PAHs	**9042**	< LOD	**90.2**

### Elemental Analysis in the 100% WSF of Oil Recovered in 2019 and 2021

Among the 28 elements identified in the samples, 24 were above the detection limit in the oil recovered in 2019, while 13 were detected in the oil recovered in 2021 (Table 2). Heavy metals exhibited the highest concentrations in the WSF of the oil recovered in 2019 and 2021. Zn, Sr, and Cr presented the highest concentration in the WSF of 2019, while Zr, Sn, Al, and Fe presented the highest concentration in the WSF of 2021 (Table 2).

**Table 2:** Concentration (mg/L) of metals in the 100% soluble fraction (WSF) used in the chronic test of oils recovered in the Marine Protected Area Costa dos Corais in 2019 and 2021

	2019	2021	CONAMA 357/2005
			Conservation units
Light metals		mg/L	
(Rb)	0.308	0.149	–
(Ba)	0.136	0.072	1
∑	0.444	0.221	
*Transition metals*			
(Y)	0.002	< LOD	–
(Fe)	< LOD	1.121	0.3
(Mn)	0.023	< LOD	0.1
(Ti)	< LOD	0.035	–
∑	0.025	1.156	–
*Rare earth*			
(Ce)	0.0025	0.0017	–
(La)	0.0023	0.001	–
∑	0.0048	0.0027	
*Heavy metals*			
(Zn)	9.52	0.169	0.09
(Al)	< LOD	83.51	1.5
(Sr)	8.581	11.842	–
(Pb)	0.120	< LOD	0.01
(Cu)	0.117	0.048	0.005
(Bi)	0.113	< LOD	–
(Se)	0.99	0.164	0.01
(Mo)	0.055	< LOD	–
(Ag)	0.043	< LOD	0.005
(Cd)	0.039	< LOD	0.005
(Ni)	0.027	< LOD	0.025
(Cr)	10.87	< LOD	0.05
(Zr)	0.009	112.51	–
(Au)	0.008	< LOD	–
(V)	0.003	< LOD	–
(Hg)	0.002	< LOD	0.0002
(Sn)	0.002	19.44	–
(Nb)	0.002	< LOD	–
(W)	0.002	< LOD	–
∑	**30.47**	**227.5**	

### Toxicity Assays 2019 and 2021

**Fig. 1:**
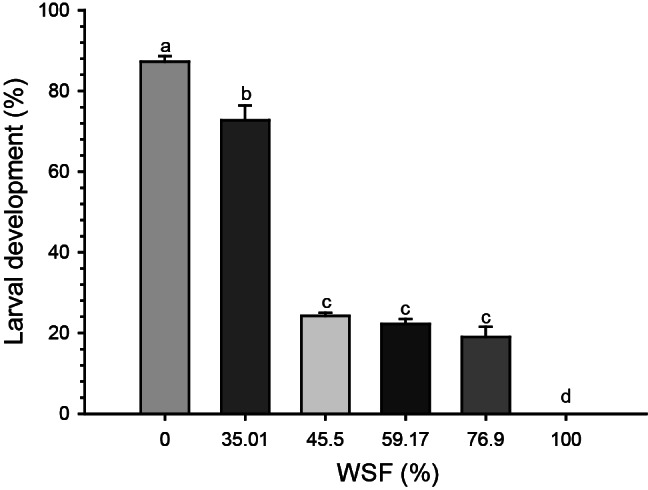
Larval development (%) of sea urchin larvae (*Echinometra lucunter*) exposed to different concentrations of the water-soluble fraction (WSF) of oil recovered in the Costa dos Corais Environmental Protection Area in 2019. Different letters indicate statistical differences between groups (*p* < 0.05)

All WSF concentrations from the 2019 recovered oil exhibited toxicity to *E. lucunter*, causing delays or inhibition in embryonic–larval development (Fig. 1). Specifically, WSF concentrations of 35.01%, 45.5%, 59.17%, and 76.9% delayed development by 1.2-, 3.6-, 3.9-, and 4.6-fold, respectively, compared to the control (0%) and compared to the 35.01% WSF, 45.5%, 59.17%, and 76.9% further reduced embryonic–larval development by 3-, 3.3-, and 3.8-fold, respectively. The 100% WSF completely inhibited embryonic–larval development. The Lowest Observed Effect Concentration (LOEC) was 35.01%. Therefore, the No Observed Effect Concentration (NOEC) was not determined from the tested concentrations, and it was > 35.01%. The calculated EC_50_ was 39.45% (95% Confidence Interval 37.04–42.01%).

**Fig. 2:**
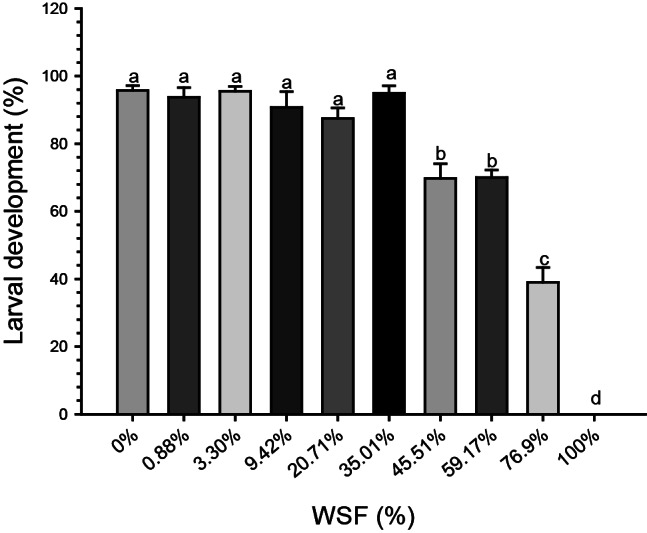
Larval development (%) of sea urchin larvae (*Echinometra lucunter*) exposed to different concentrations of the water-soluble fraction (WSF) of oil recovered in the Costa dos Corais Environmental Protection Area in 2021. Different letters indicate statistical differences between groups (*p* < 0.05)

For the oil recovered in 2021, WSF concentrations of 45.51% and 59.17% delayed embryonic–larval development by 1.3-fold compared to the control (0%) and to lower concentrations (0.88%, 3.30%, 9.42%, 20.71%, and 35.01%). The 76.9% WSF reduced embryonic–larval development by 2-fold compared to the control and the lower concentrations (0.88%, 3.30%, 9.42%, 20.71%, and 35.01%), and by 1.57-fold compared to 45.51% and 59.17%. Complete inhibition of embryonic–larval development was observed at the 100% WSF concentration (Fig. 2). The Lowest Observed Effect Concentration (LOEC) was 45.51%, and the No Observed Effect Concentration (NOEC) was 35.01%. The calculated EC_50_ was 65.42% (95% Confidence Interval: 61.09–70.05%).

## Discussion

The 100% WSF of the oil recovered in 2019 exhibited a relatively low concentration of PAHs (9042 ng/L) compared to WSF from oils in other regions (Rodrigues et al. 2010; Santana et al. 2025). However, despite this, the PAHs concentration exceeded the Brazilian water quality criteria established for class I saline waters (aquatic environments in fully protected conservation units) (CONAMA nº357 2005). Among the analyzed compounds, dibenzo[a, h]anthracene levels were approximately 60 times higher than the limits set by Brazilian legislation, followed by indeno[1,2,3-cd]pyrene (30 times), benzo[a]anthracene, chrysene, benzo[b]fluoranthene, and benzo[a]pyrene (20 times), and benzo[k]fluoranthene (8 times). Similarly, Santana et al. (2025) reported that the concentrations of these seven PAHs exceeded Brazilian legal limits by 10 to 100 times in the WSF of oil recovered from beaches (Trancoso and Massarandupió, Bahia) affected by the same oil spills in Brazil. In addition to the comparison with Brazilian legislation (CONAMA 357/2005), the NOAA Screening Quick Reference Tables (SQuiRTs; Buchman 2008) was consulted. These guidelines compile threshold effect levels (TELs) and probable effect levels (PELs) for various contaminants in aquatic environments. When comparing the present study results with these international benchmarks, PAHs such as phenanthrene, anthracene, and dibenzo[a, h]anthracene were present at levels that surpass the SQuiRT thresholds, supporting their role as major toxic agents in early developmental stages of *E. lucunter*. In contrast, the 2021 WSF showed concentrations below detection limits for PAHs, consistent with reduced toxicity.

Notably, while naphthalene and its alkylated homologs often dominate PAH concentrations in oil WSF from other sources (Rodrigues et al. 2010; Santana et al. 2025), the 100% WSF from the 2019 recovered oil in this study contained all 16 analyzed low- and high-molecular-weight PAHs. This broad PAH profile, encompassing compounds like naphthalene, fluoranthene, phenanthrene, fluorene, and pyrene, as also identified by Soares et al. (2021) in spill samples, contrasts with studies like Rodrigues et al. (2010), who found a more limited set (naphthalene, fluorene, phenanthrene) in Brazilian heavy oil WSF. While petrogenic PAHs typically show a predominance of low-molecular-weight compounds (Wang et al. 1999), the high PAH concentrations in the 2019 oil, exceeding Brazilian regulatory limits (CONAMA nº357 2005), indicated minimal weathering (e.g., evaporation, dissolution, biodegradation) during its transport to shore. In contrast, 2 years post-spill (2021), no PAHs were detectable in the WSF, suggesting significant weathering processes such as evaporation, emulsification, natural dispersion, dissolution, microbial degradation, and photooxidation had occurred (Lobão et al. 2022). This is consistent with the mechanism proposed by Perugini et al. (2007), whereby PAHs with low water solubility (often higher-molecular-weight) tend to sediment with particulate matter, while more soluble, lower-molecular-weight PAHs remain in the water column and undergo more rapid weathering.

Multi-elemental analysis of the 100% WSF from oils recovered in 2019 and 2021 revealed the presence of twenty-eight elements, thirteen of which (Al, Ba, Cd, Pb, Cu, Cr, Fe, Mn, Hg, Ni, Ag, Se, and Zn) are regulated by the Brazilian agency CONAMA (2005). The occurrence of such metals in petroleum is intrinsically linked to the geological location of its formation, where metals from the surrounding rock composition can dissociate in water via rock pores and subsequently be absorbed by the oil (Akpoveta and Osakwe 2014). The differing metal profiles in the 2019 and 2021 WSF indicate that while some elements persisted in the WSF over time, others were no longer detected, and a few (e.g., Fe, Ti, Al), previously below detection or quantification limits in 2019, were detected in the 2021 recovered oil. Notably, Zn, Cu, and Se consistently exceeded Brazilian regulatory limits in both years, suggesting their high solubility in oil, possible continuous inputs from natural or anthropogenic sources (e.g., sediment resuspension, coastal runoff, vessel discharges) (Lemly 2004). In addition to the comparison with Brazilian legislation (CONAMA 357/2005), when comparing metal(oid)s results with NOAA Screening Quick Reference Tables (SQuiRTs; Buchman 2008), several metal(oid)s detected in the 2019 WSF exceeded the TEL values (e.g., Zn, Cu, and Se), reaching concentrations within or above the range where adverse effects on aquatic organisms are frequently observed. The reduction in the number of metals exceeding these limits, from nine in 2019 to five in 2021, may indicate natural attenuation processes such as precipitation, adsorption onto particles, reduced bioavailability due to chemical speciation changes, or participation in enzymatic activity, similar to observations following the 1991 Gulf War where residual metal concentrations were lower compared to pre-event analyses (Hirose 2006; Freije 2015). The presence of Al and Fe in the 2021 WSF, but not in 2019, could be related to the incorporation of particulate material from the environment over time (Ramasamy et al. 2019; Revel et al. 2025). Conversely, the disappearance of Pb, Ag, Cd, Ni, Cr, and Hg from the WSF might suggest their removal through complexation and the formation of insoluble precipitates, reducing bioavailability. However, historical contamination events like those in the Persian Gulf have shown that decreased environmental metal concentrations could result from the bioaccumulation in local biota (Freije 2015). To further explore the origin of metals, we compared all concentrations against Al and Fe, which are widely used as lithogenic tracers. The scatterplots (Supplementary Material, S4) show that in the 2021 WSF, several elements (e.g., Zr, Sn, Sr, Zn, Cu, Se) deviated strongly from Al and Fe, indicating enrichment beyond natural terrigenous sources, and supporting their anthropogenic origin. For the 2019 WSF, Al and Fe were below detection limits, preventing scatterplot analysis; however, the elevated concentrations of Zn, Cr, and Pb above regulatory criteria are consistent with the same interpretation. A significant concern is the high concentration of several metal(oid)s for which Brazilian environmental legislation established no regulatory limits (CONAMA 2005) (Rb, Ti, Bi, Mo, Nb, Sr, V, Y, La, Ce, W, Zr). Some of these, such as V and Mo, are commonly associated with petroleum and may indicate natural oil sources, with their concentrations potentially influenced by adsorption to other substances (Liu et al. 2012). Others, including La, Ce, Y, and Nb, may be linked to anthropogenic sources such as industrial waste or the presence of element-rich soils, as reported in studies on waste dumping in the Persian Gulf (Naser 2013).

In the present study, the WSF of the oil collected in Northeastern Brazil in 2019 and 2021 significantly impaired embryonic–larval development in *Echinometra lucunter*. The 2019 WSF, even at its lowest tested concentrations, produced developmental delays and abnormalities, consistent with high PAHs and metal(oid)s concentrations. Badri et al. (2024) showed that sea urchins (*Echinometra mathaei*) exposed to PAHs accumulated 913.7 ng/g of naphthalene and 2047.8 ng/g of phenanthrene in their tissues, while and Suzuki (2020) reported significant morphological alterations in *Hemicentrotus pulcherrimus* pluteus larvae exposed to PAHs. These findings corroborate the sensitivity of early developmental stages to PAHs exposure and align with the broad PAHs profile observed in the 2019 WSF, where all 16 analyzed compounds were present at concentrations exceeding Brazilian regulatory limits (CONAMA 2005).

By 2021, PAHs were no longer detectable in the WSF, reflecting extensive weathering processes (e.g., evaporation, dissolution, microbial degradation, photooxidation). However, toxicity persisted at higher LOEC values and was associated with metals above detection and regulatory limits. The differing metal profiles between 2019 and 2021 highlight that while some elements persisted, others disappeared, and new ones (e.g., Fe, Ti, Al) emerged, likely reflecting oil’s interaction with environmental matrices. Notably, Zn, Cu, and Se consistently exceeded regulatory limits in both years, supporting their role as major toxic agents. Our results are consistent with toxicity thresholds previously reported in the literature. Fernández and Beiras (2001) found EC_50_ values of 21.9 µg/L for Cu, 66.8 µg/L for lead, 509 µg/L for Cd, and 9240 µg/L for Ni in sea urchin embryos. In *Sterechinus neumayeri*, Cu was again the most toxic metal (EC_50_ = 11.4 µg/L), whereas Cd only impacted development at concentrations above 2000 µg/L (EC_50_ = 6900 µg/L). Zinc has also shown EC_50_ values of 2230 and 326.8 µg/L in Antarctic Sea urchins (King and Riddle 2001). Similarly, Bielmyer et al. (2005) reported LOECs for Cu (11 µg/L), Ag (6 µg/L), Ni (15 µg/L), and Se (26 µg/L) in *Diadema antillarum* embryos. The concentrations of these metals in 2019 WSF exceeded or were within the same order of magnitude as these effect thresholds, reinforcing their likely contribution to the developmental impairments observed. This indicates that, although overall toxicity decreased after weathering, weathered oil residues may act as vectors of metallic contamination, altering the profile of soluble elements over time and sustaining ecotoxicological risks. These comparative toxicity values reinforce that the concentrations of PAHs and metal(oid)s detected in the WSF analyzed here are within ranges known to impair early development in sea urchins, supporting the conclusion that both contaminant classes likely contributed to the observed effects.

## Conclusion

The marked reduction in embryo–larval development observed in *E. lucunter* clearly demonstrates the toxicity of the oil spilled along the Northeastern Brazilian coast in 2019, underscoring the potential for adverse effects on other marine and coastal organisms at the time of the event. Although toxicity decreased after 2 years, likely due to oil weathering (with PAHs falling below detection limits and a reduction in certain metal(loid) concentrations), the 2021 WSF still caused developmental delays. By demonstrating the persistence of oil-related toxic effects, this study provides valuable insights for both the scientific community and environmental management and reinforces the urgent need for more effective techniques to remove contaminants within shorter timeframes.

## Supplementary Information

Below is the link to the electronic supplementary material.

Supplementary Material 1

## Data Availability

The data supporting this study’s findings are available from the corresponding author upon reasonable request.
