# Supplementary material for: Toxicity of Water-Soluble Fraction in Echinometra lucunter for the 2019 and 2021 Accidental Oil Spills on the Brazilian Coast
Source: Bull Environ Contam Toxicol. 2026 Apr 28;116(5):93. doi: 10.1007/s00128-026-04247-6 (PMC13124846; doi:10.1007/s00128-026-04247-6)

**Supplementary material**

| **S1.** Compounds, retention times, and quantification and confirmation m/z ratios used in the selective ion monitoring (SIM) mode.   \| Compound \| Retetion time (min) \| m/z \| \| \| --- \| --- \| --- \| --- \| \| Quantification \| Confirmation \| \| **Naphthalene-D8** \| **9.209** \| **136** \| ***136*, 137, 108** \| \| Naphthalene \| 9.252 \| 128 \| *128*, 127, 129 \| \| Acenaphthylene \| 12.809 \| 152 \| *152*, 151,150 \| \| **Acenaphthene-d10** \| **13.151** \| **162** \| ***162*, 164,160** \| \| Acenaphthene \| 13.226 \| 153 \| *153*, 154, 152 \| \| Fluorene \| 14.455 \| 166 \| *166*, 165, 164 \| \| **Phenanthrene-D10** \| **16.701** \| **188** \| ***188*, 187, 184** \| \| Phenanthrene \| 16.756 \| 178 \| *178*, 176, 152 \| \| Anthracene \| 16.876 \| 178 \| *178*, 176, 179 \| \| Fluoranthene \| 20.002 \| 202 \| *202*, 200, 203 \| \| Pyrene \| 20.339 \| 202 \| *202*, 200, 201 \| \| **p-Terphenyl-d14** \| **20.929** \| **244** \| ***244*, 243, 245** \| \| Chrysene \| 24.883 \| 228 \| *228*, 226, 229 \| \| **Chrysene-D12** \| **24.924** \| **240** \| ***240*, 236, 241** \| \| Benz[a]anthracene \| 25.034 \| 228 \| *228*, 226, 229 \| \| Benzo[b]fluoranthene \| 30.512 \| 252 \| *252*, 250, 253 \| \| Benzo[k]fluoranthene \| 30.694 \| 252 \| *252*, 250, 253 \| \| Benzo[a]pyrene \| 32.204 \| 252 \| *252*, 250, 253 \| \| **Perylene-D12** \| **32.440** \| **264** \| ***264*, 260, 265** \| \| Indeno[1,2,3-cd]pyrene \| 36.209 \| 276 \| *276*, 277, 274 \| \| Dibenz[a,h]anthracene \| 36.350 \| 278 \| *278*, 279, 276 \| \| Benzo[ghi]perylene \| 37.249 \| 276 \| *276*, 277, 274 \| | | | |
| --- | --- | --- | --- | --- | --- | --- | --- | --- | --- | --- | --- | --- | --- | --- | --- | --- | --- | --- | --- | --- | --- | --- | --- | --- | --- | --- | --- | --- | --- | --- | --- | --- | --- | --- | --- | --- | --- | --- | --- | --- | --- | --- | --- | --- | --- | --- | --- | --- | --- | --- | --- | --- | --- | --- | --- | --- | --- | --- | --- | --- | --- | --- | --- | --- | --- | --- | --- | --- | --- | --- | --- | --- | --- | --- | --- | --- | --- | --- | --- | --- | --- | --- | --- | --- | --- | --- | --- | --- | --- | --- | --- | --- | --- | --- | --- | --- | --- |
| *m/z: Mass-to-charge ratio. |  |  |  |
| *SIM stands for: Selected Ion Monitoring. |  |  |  |

**S2.** Compounds, parameters of the analytical curves, limits of quantification (LOQ) and detection (LOD), and the coefficient of determination (R²) for the analysis of the oil WSF samples (2019 and 2021).

| **Compound** | Linear range | LOQ | LOD | r^2^ |
| --- | --- | --- | --- | --- |
|  | ng L^-1^ | | |  |
| **Naphthalene** | 100 to 2000 | 79 | 26 | 0.992 |
| **Acenaphthylene** | 100 to 2000 | 41 | 13 | 0.990 |
| **Acenaphthene** | 100 to 2000 | 37 | 12 | 0.990 |
| **Fluorene** | 100 to 2000 | 21 | 7 | 0.991 |
| **Phenanthrene** | 100 to 2000 | 97 | 32 | 0.989 |
| **Anthracene** | 100 to 2000 | 26 | 9 | 0.991 |
| **Fluoranthene** | 100 to 2000 | 94 | 31 | 0.988 |
| **Pyrene** | 100 to 2000 | 35 | 12 | 0.990 |
| **Chrysene** | 100 to 800 | 90 | 30 | 0.993 |
| **Benz[a]anthracene** | 100 to 800 | 33 | 11 | 0.991 |
| **Benzo[b]fluoranthene** | 100 to 800 | 96 | 32 | 0.991 |
| **Benzo[k]fluoranthene** | 100 to 800 | 31 | 10 | 0.992 |
| **Benzo[a]pyrene** | 100 to 800 | 33 | 11 | 0.992 |
| **Indeno[1,2,3-cd]pyrene** | 100 to 800 | 28 | 9 | 0.990 |
| **Dibenz[a,h]anthracene** | 100 to 400 | 10 | 3 | 0.994 |
| **Benzo[ghi]perylene** | 100 to 800 | 60 | 20 | 0.988 |

Internal standard (IS): p-terphenyl-D14 at a concentration of 1 ng L^-1^.

**S3.** Limits of quantification (LOQ) and detection (LOD) for the metals analysis of the 2019 and 2021 oil WSF.

| **Elements** | 2019 | | 2021 | |
| --- | --- | --- | --- | --- |
|  | LOD | LOQ | LOD | LOQ |
|  | μg L^-1^ | | | |
| **Al** | 0.07159 | 0.07159 | 0.11613 | 0.31296 |
| **Ti** | 0.02510 | 0.02510 | 0.06284 | 0.17004 |
| **V** | 0.00401 | 0.00401 | 0.00935 | 0.02490 |
| **Cr** | 0.01006 | 0.01991 | 0.01176 | 0.02989 |
| **Mn** | 0.05600 | 0.15120 | 0.04400 | 0.09900 |
| **Fe** | 0.54228 | 0.82248 | 0.30393 | 0.81472 |
| **Fe** | 0.04681 | 0.04681 | 0.38148 | 1.02259 |
| **Ni** | 0.04681 | 0.04681 | 0.11250 | 0.27261 |
| **Cu** | 0.23011 | 0.29533 | 0.25480 | 0.08410 |
| **Zn** | 0.00013 | 0.00013 | 0.22426 | 0.60927 |
| **As** | 0.03938 | 0.08665 | 0.11869 | 0.28014 |
| **Se** | 0.01503 | 0.01503 | 0.26072 | 0.52020 |
| **Rb** | 0.38865 | 0.44757 | 0.00986 | 0.02629 |
| **Sr** | 0.00402 | 0.00402 | 0.00293 | 0.00794 |
| **Y** | 0.00210 | 0.00210 | 0.00220 | 0.00452 |
| **Zr** | 0.00206 | 0.00206 | 0.03561 | 0.07838 |
| **Nb** | 0.00021 | 0.00021 | 0.01857 | 0.04602 |
| **Mo** | 0.03840 | 0.08640 | 0.15903 | 0.40583 |
| **Ag** | 0.00058 | 0.00058 | 0.02555 | 0.06231 |
| **Cd** | 0.01851 | 0.01851 | 0.08460 | 0.18483 |
| **Sn** | 0.00157 | 0.00157 | 0.03618 | 0.09271 |
| **Ba** | 0.00492 | 0.00492 | 0.11453 | 0.30480 |
| **La** | 0.00016 | 0.00016 | 0.00204 | 0.00340 |
| **Ce** | 0.00015 | 0.00015 | 0.00089 | 0.00234 |
| **W** | 0.00037 | 0.00037 | 0.00713 | 0.01667 |
| **Au** | 0.00045 | 0.00045 | 0.02085 | 0.05496 |
| **Hg** | 0.01275 | 0.01275 | 0.10507 | 0.28560 |
| **Hg** | 0.01221 | 0.01221 | 0.11980 | 0.31551 |
| **Pb** | 0.01248 | 0.01248 | 1.24177 | 3.15621 |
| **Bi** | 0.01942 | 0.01942 | 0.50712 | 1.29233 |

**S4.** Scatterplots of metal concentrations in the 100% water-soluble fraction (WSF) of oil recovered in 2021 plotted against lithogenic tracers **Al** (left) and **Fe** (right), in log–log scale.


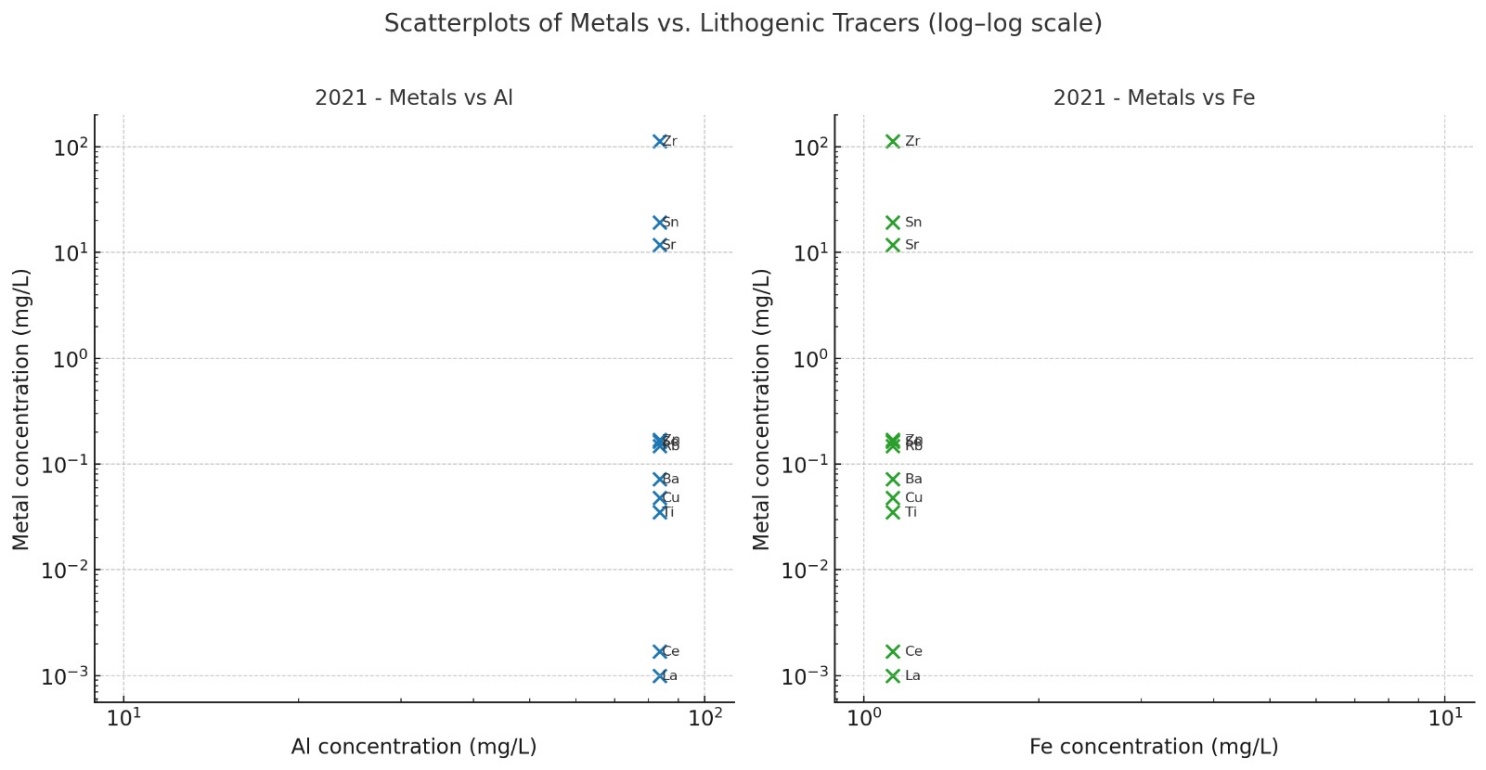

Supplement: Supplementary file 1 — Supplementary Material 1 [file 128_2026_4247_MOESM1_ESM.docx]
